# Non-Neuraxial Chest and Abdominal Wall Regional Anesthesia for Intensive Care Physicians—A Narrative Review

**DOI:** 10.3390/jcm13041104

**Published:** 2024-02-15

**Authors:** Sascha Ott, Lukas M. Müller-Wirtz, Gokhan Sertcakacilar, Yasin Tire, Alparslan Turan

**Affiliations:** 1 Department of Outcomes Research, Anesthesiology Institute, Cleveland Clinic, Cleveland, OH 44195, USA; muellel2@ccf.org (L.M.M.-W.); g.sertcakacilar@saglik.gov.tr (G.S.); yasin.tire@saglik.gov.tr (Y.T.); turana@ccf.org (A.T.); 2 Deutsches Herzzentrum der Charité—Medical Heart Center of Charité and German Heart Institute Berlin, Department of Cardiac Anesthesiology and Intensive Care Medicine, Augustenburger Platz 1, 13353 Berlin, Germany; 3 Charité—Universitätsmedizin Berlin, Corporate Member of Freie Universität Berlin, Humboldt-Universität zu Berlin, Charitéplatz 1, 10117 Berlin, Germany; 4 Department of Anaesthesiology, Intensive Care and Pain Therapy, Saarland University Medical Center, Saarland University Faculty of Medicine, 66424 Homburg, Germany; 5 Department of Anesthesiology and Reanimation, Bakırköy Dr. Sadi Konuk Training and Research Hospital, 34147 Istanbul, Turkey; 6 Department of Anesthesiology and Reanimation, Konya City Hospital, University of Health Science, 42020 Konya, Turkey; 7 Department of General Anesthesiology, Anesthesiology Institute, Cleveland Clinic, Cleveland, OH 44195, USA

**Keywords:** regional anesthesia, peripheral nerve blocks, chest wall blocks, abdominal wall blocks, airway blocks, ICU

## Abstract

Multi-modal analgesic strategies, including regional anesthesia techniques, have been shown to contribute to a reduction in the use of opioids and associated side effects in the perioperative setting. Consequently, those so-called multi-modal approaches are recommended and have become the state of the art in perioperative medicine. In the majority of intensive care units (ICUs), however, mono-modal opioid-based analgesic strategies are still the standard of care. The evidence guiding the application of regional anesthesia in the ICU is scarce because possible complications, especially associated with neuraxial regional anesthesia techniques, are often feared in critically ill patients. However, chest and abdominal wall analgesia in particular is often insufficiently treated by opioid-based analgesic regimes. This review summarizes the available evidence and gives recommendations for peripheral regional analgesia approaches as valuable complements in the repertoire of intensive care physicians’ analgesic portfolios.

## 1. Introduction

In the mid-1990s, the American Pain Society started promoting pain as the fifth vital sign. Emphasizing the significance of pain management, current data reveal that nearly 50% of patients in critical care settings experience pain even at rest, with this number escalating when pain during movements or procedures is considered [1,2].

Pain is well known to elicit discomfort and contribute to acute morbidity for critically ill patients [3]. Inadequate pain control might lead to harmful effects on the respiratory system, for instance, hypoventilation, insufficient ability to cough properly, subsequent atelectasis, and possibly pneumonia [4]. Effects on the cardiovascular system include tachycardia and increased myocardial oxygen demand. Among non-cardiac surgery patients, pain after surgery was associated with myocardial injury, impaired pulmonary function, ileus, thromboembolism, delayed wound healing, and an increased risk of infection [5,6,7,8]. Furthermore, insufficiently treated acute pain may facilitate the development of chronic pain. Several studies report prevalence rates between 33 and 56% for chronic pain at six months and up to two years after patients’ ICU discharge [9,10,11]. After coronary artery bypass graft (CABG) surgery, up to 56% develop chronic postsurgical pain [12,13].

Regional anesthesia (RA) as part of multi-modal approaches to pain management strategies has proved to significantly reduce the need for opioids in the perioperative setting [14]. Opioids represent an established and indispensable cornerstone in the analgesic and sedative treatment of ICU patients and facilitate the feasibility of many ICU-related procedures. Nevertheless, especially in ICU patients, opioids can contribute to undesirable side effects like constipation and ileus. In this context, multi-modal analgesic approaches represent an opportunity to reduce the need for opioids and thereby might contribute to decreased associated side effects. However, such multi-modal approaches are not widely used in the intensive care unit (ICU) setting, and mono-modal opioid-based analgesic strategies represent the standard for managing moderate to severe pain [15,16,17]. Against this background, and supported by a great amount of evidence from clinical trials, the American Pain Society and the American Society of Anesthesiologists identified a multi-modal treatment approach with RA as one major and key element [18,19]. The most common regional methods are neuraxial procedures; however, peripheral nerve blocks (PNBs) and fascial plane blocks (FPBs) might be advantageous in critically ill patients, where possible side effects of neuraxial procedures are feared. PNBs and FPBs for thoracic or abdominal pain control might particularly interest critical care physicians in this context. A recent meta-analysis showed the beneficial effect of PNB in a multi-modal approach compared to conventional pain management strategies in the first 24 h in patients with rib fractures [20]. 

Whether in surgical patients where regional techniques are not part of the initial anesthetic concept or in non-surgical patients with insufficient systemic pain control, an escalation of the analgesic strategy using PNBs/FPBs can be a valuable tool either for the critical care provider itself or when provided by an anesthesiologist. Current evidence mainly comes from the perioperative setting, and data gained in the ICU population are predominantly limited to rib fractures and acute pancreatitis. This review summarizes the available evidence and gives an overview of regional anesthesia techniques for the thorax, abdomen, and airway for intensive care physicians that provide a favorable safety profile in most patients.

## 2. Special Considerations for Regional Anesthesia in Critically Ill Patients

Critically ill patients present unique challenges compared to otherwise healthy individuals receiving a perioperative PNB/FPB. Thus, it might be challenging to position patients properly for convenient access. Tissue edema, multiple other catheters, and hemodynamic or bleeding instabilities further complicate regional anesthesia procedures [21]. There are no data available comparing single shots blocks versus continuous application with a catheter in ICU patients. However, when facing the aforementioned challenges, it seems reasonable to consider choosing a catheter-based continuous approach rather than repeated single shot applications. 

Over the past decade, ultrasound-guided (USG) methods have become the standard for regional anesthesia techniques, providing additional benefits for ICU patients [22]. Especially when anatomically conditions are challenging due to tissue edema or suboptimal positioning, USG techniques are of inestimable value, especially when compared to nerve stimulation guidance, which might be attenuated due to neuromuscular weakness in critically ill patients [23]. 

The underlying condition of critically ill patients influences the pharmacokinetics and pharmacodynamics on multiple levels, including alterations in the volume of distribution and/or drug metabolization. Thus, due to renal or liver insufficiency, the elimination of local anesthetics is likely to be altered, and the risk of Local Anesthetic Systemic Toxicity (LAST) may be elevated [24]. Early signs of LAST are neurological symptoms such as perioral numbness, tinnitus, agitation, and confusion, which might not be evaluated in sedated ICU patients. This is why considering cardiac symptoms such as arrhythmias and extrasystoles becomes more critical. In this context, USG techniques allow a more accessible, more reliable, and more precise identification of anatomical structures and the application of lower doses of LA to prevent LAST [25].

Another aspect is that mechanical ventilation, subsequently changing intrathoracic pressures and patient positioning, influences the spread of epidural or spinal techniques, possibly aggravating their controllability [26]. PNBs and FPBs can be advantageous here again.

Infections, especially catheter-related infections, are a significant concern in cohorts of ICU patients. The prevalence of septic patients is significantly higher in the ICU compared to other wards. Particularly in these patients, there might be concerns regarding the safety of additional invasive procedures, especially for continuous application techniques with regional anesthesia catheters. The overall risk for regional anesthesia catheter-related infections in the perioperative setting is low [27,28]. In the Third National Audit Project Report, the incidence of epidural abscesses in the perioperative period was approximately 1 in 47,000, with an incidence of harm from vertebral abscess of 1 in 88,000 [29]. The incidence of bacterial meningitis was lower than 1 in 200,000. These incidences, however, are based on perioperative data in non-immunocompromised, non-septic patients. On the one hand, no guidance from evidence is available for the conduction of PNBs/FPBs in ICU patients or when systemic sepsis is present. Data coming from other typical ICU-related catheters, such as central venous lines, suggest that the risk of an infection is modified by multiple risk factors such as the immune status of the patient, catheterization duration, and length of ICU stay [30]. However, these data might not be uncritically assigned to regional anesthesia catheters. Still, it would be safe to assume that the PNB/FPB-associated risks are lower than for neuraxial procedures. The alternative of an opioid-based analgesic approach is potentially not risk-free either. Opioids are known to be immunomodulating, and the associated influence on the immune system is not fully understood [31]. Thus, unnecessary high dosages of opioids related to mono-modal approaches may have relevant implications for critically ill patients [32]. 

The concurrent use of anticoagulants limits the applicability of regional anesthesia techniques. While there are no official recommendations regarding the utilization of regional anesthesia in ICU patients, it seems reasonable to adhere to existing guidelines and recommendations for regional anesthesia in anticoagulated patients when contemplating such procedures [33]. Accordingly, neuraxial and deep blocks are contraindicated in anticoagulated patients, whereas plexus or peripheral techniques are feasible with respect to visibility and the possibility of local compression if needed. Critically ill patients receive anticoagulants as part of their usual treatment to prevent deep vein thrombosis. When scheduling regional anesthesia, it is essential to align the timing of the procedure with anticoagulant administration to avoid hematomas. USG is particularly beneficial in this context. 

The most commonly used regional anesthesia technique in critical care medicine is epidural anesthesia [21]. However, when facing specific complications and contraindications of neuraxial procedures, PNBs and FPBs might offer a valuable alternative in ICU patients where neuraxial methods are considered inappropriate or unsafe. In addition to advantages in terms of bleeding and anticoagulation management, PNBs/FPBs have been shown to have a safer hemodynamic profile when compared to epidural anesthesia [34,35].

## 3. Peripheral Nerve Blocks for the Chest Wall

Pectoralis nerve blocks, serratus anterior plane blocks, erector spinae plane blocks, paravertebral blocks, and parasternal blocks can provide valuable supplements in the portfolio of multi-modal analgesic approaches in chest wall pain. While the indication spectrum and covered areas for some of these blocks might overlap, the feasibility and, thereby, the choice of one of these blocks might differ according to the specifics of the concrete ICU patient. 

### 3.1. Pectoralis Nerve Block

#### 3.1.1. How to Perform the Block

**Figure 1:**
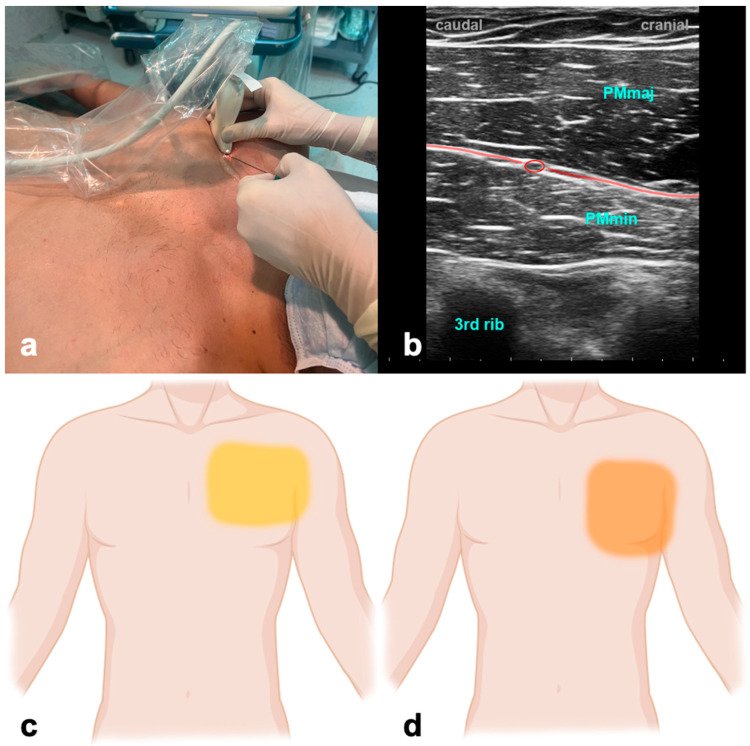
Pectoralis nerve block. (**a**) Transducer positioning. (**b**) Ultrasound anatomy of PECS I: PMmaj—pectoralis major muscle; PMmin—pectoralis minor muscle; red circle—pectoral branch of thoracoacromial artery; red line target space for PECS I block. (**c**) Spread of PECS I. (**d**) Spread of PECS II.

The patient is supine with the arm abducted at 90 degrees or at the side. The ultrasound probe is positioned under the clavicle at the midclavicular line to locate the axillary artery and vein beneath the pectoralis major and minor muscles. Subsequently, the probe is shifted in a distal and lateral direction while visualizing the ribs as a reference point, moving it toward the axilla (Figure 1). At the level of the third and fourth rib, the major and minor pectoralis muscle and the thoracoacromial artery’s pectoral branch can be identified. For PECS I, the needle is inserted in-plane in a medial to lateral direction until the tip enters the fascial plane between the pectoralis major and minor muscle. To achieve PECS II, the needle is then further advanced to the plane between the pectoralis minor and serratus muscles. Volumes of LA are indicated in Table 1.

#### 3.1.2. Evidence and Indications

No studies have addressed PECS blocks in ICU patients, and the available evidence in the perioperative setting is inconsistent. However, PECS II was beneficial compared to systemic analgesia alone in mastectomy [36,37,38] but failed to improve postoperative analgesia or cumulative opioid consumption after robotically assisted mitral valve repair [39]. An ongoing trial evaluates the impact of PECS II blockade in patients undergoing minimally invasive cardiac surgery [40]. PEC blocks affect the intercostobrachial and intercostal nerve distribution, at T3–T6, and the long thoracic nerve, which together innervate the anterolateral chest and adjacent axilla.

#### 3.1.3. Why the ICU Physician Could Love It

Postoperative pain resulting from thoracic and breast surgeries often poses challenges, impacting patients’ ability to take deep breaths and contributing to substantial morbidity. Performing PECS blocks is a favorable option due to the accessible anatomy and the relative safety afforded by the ribs shielding against an inadvertent puncture of the pleura.

### 3.2. Serratus Anterior Plane Block

#### 3.2.1. How to Perform the Block

**Figure 2:**
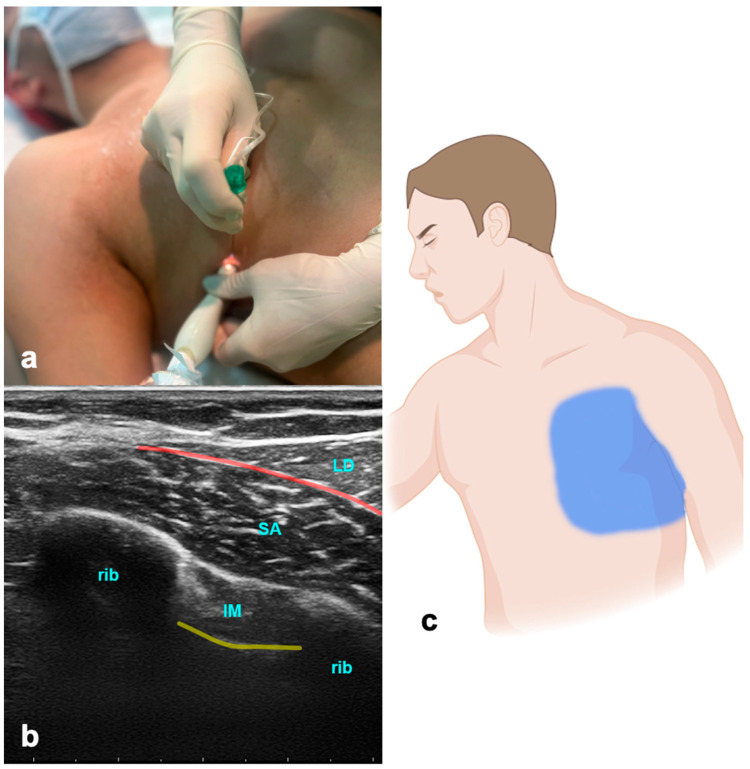
Serratus anterior plane block. (**a**) Transducer positioning. (**b**) Ultrasound anatomy of SAPB: LD—latissimus dorsi muscle; SA—serratus anterior muscle; IM—intercostal muscle; yellow line—pleura; red line—target space for SAPB. (**c**) Spread of SAPB.

The ultrasound transducer is positioned in the mid-axillary line in the transverse plane at the level of the fifth rib (Figure 2). The USG landmarks are the ribs, pleural lines, and serratus anterior and latissimus dorsi muscles. The needle is advanced in-plane towards the fifth rib, and the LA is applied above the serratus muscle for a superficial SAPB and anteriorly to the rib and deep into the serratus muscle for a deep SAPB. Volumes of LA are indicated in Table 1.

#### 3.2.2. Evidence and Indications

The serratus anterior plane block (SAPB) has been used effectively in managing pain in rib fractures [41], thoracic surgery [42], breast surgery, and post-mastectomy pain syndrome [43,44]. Data from a small study suggest that the SAPB is equally suitable for managing pain in patients with rib fractures compared to epidural and paravertebral blocks [45]. Another study showed a continuous SAPB to be similarly effective in pain control compared to intravenous fentanyl in rib-fractured patients, with superior early mobilization and shorter ICU lengths of stay [46]. In a recent meta-analysis, the SAPB was shown to reduce both pain scores and 24 h postoperative opioid consumption [47]. The SAPB is a safe and effective alternative for thoracic epidural analgesia (TEA) for postoperative analgesia after thoracotomy [48]. However, the erector spinae plane block (ESPB) was superior to the SAPB in the pain management of patients with lung cancer undergoing posterolateral thoracotomy [49]. The block effectively anesthetizes the lateral cutaneous branches of the intercostal nerves, which emerge between the layers of muscles. Additionally, it provides anesthesia to the long thoracic, thoracodorsal, and intercostobrachial nerves and thereby may be beneficial in pain originating from intercostal drainages—a common source of pain in ICU patients.

#### 3.2.3. Why the ICU Physician Could Love It

The SAPB presents notable advantages over epidural or paravertebral blocks, particularly in the context of rib fractures. Aside from distinct issues and positioning challenges associated with other blocks, the SAPB offers a relatively simpler procedure, making it a more accessible and practical choice for effective pain management.

### 3.3. Erector Spinae Plane Block

The erector spinae plane block (ESPB) is a relatively new PNB with rapidly growing popularity because it is easy to perform and offers a broad scope. The ESPB can be applied as a single block or continuous analgesic approach, offering a reliable and safer alternative to neuraxial procedures [50,51,52]. It has been shown that the LA applied due to an ESPB spreads to the paravertebral space, the neural foramina, and partially to the epidural space [53]. It can be performed on different levels and applies to thoracic and abdominal procedures.

#### 3.3.1. How to Perform the Block

**Figure 3:**
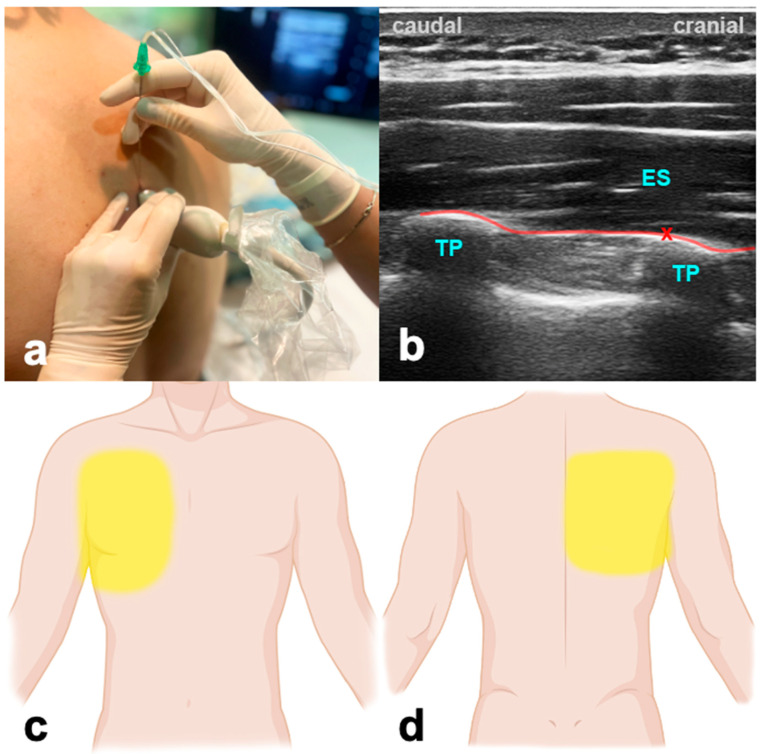
Erector spinae plane block. (**a**) Transducer positioning. (**b**) Ultrasound anatomy of ESPB: ES—erector spinae muscle; TP—transverse process; red cross—target point for the injection at the edge of the TP; red line—target space for ESPB. (**c**) Frontal spread of ESPB. (**d**) Dorsal spread of ESPB.

The patient can be in a sitting, supine, prone, or lateral decubitus position. The ultrasound probe is initially positioned longitudinally over the ribs, in the middle between the scapula and spine (Figure 3). The probe is advanced medially until first the costotransverse junction and, subsequently, the transverse process appears. The latter can be differentiated from ribs due to its more superficial, wider, and rectangular shape. The needle is inserted in a cranio-caudal direction in an in-plane technique targeting the transverse process until the tip reaches contact. LA is given to the plane between the transverse process of the thoracic or lumbar vertebra and the anterior fascia of the erector spinae muscles. Volumes of LA are indicated in Table 1.

#### 3.3.2. Evidence and Indications

Although the ESPB is a relatively new fascial block, there is much data available; however, most studies do not focus primarily on the ICU population. Plenty of data in cardiac surgery show that the ESPB provides opioid-sparing perioperative analgesia, facilitates early mobilization and extubation, allows earlier ICU discharge, and is superior in a multi-modal approach compared to mono-modal intravenous strategies [54,55,56,57,58]. The ESPB contributes to early recovery after cardiac surgery (ERACS) pathways in this context [59]. In addition, the ESPB is beneficial in reducing chronic postsurgical pain in CABG patients [60]. Beyond that, the ESPB is a valuable, low-risk, and high-success-rated tool in patients with thoracic trauma and rib fractures [61,62]. In abdominal surgery, the ESPB offers a variety of scopes. Compared to the transabdominal plane block, the bilateral ESPB was a more feasible and effective intra- and postoperative analgesia method in patients undergoing laparoscopic bariatric surgery [63]. A recent publication showed a significant reduction in intra- and postoperative opioid consumption, a major trigger for postoperative constipation and ileus [64]. 

#### 3.3.3. Why the ICU Physician Could Love It

The ESPB can prove to be a valuable tool during the weaning and extubation of patients following thoracic procedures, especially when challenges arise due to inadequate pain control. This is particularly relevant in cases where achieving optimal pain management compromises the patient’s ability for adequate spontaneous breathing efforts, such as in lung transplant patients [65]. Furthermore, the EPSB provides a superior safety profile regarding the coagulation status of the patients when compared to neuraxial blocks.

### 3.4. Paravertebral Block

#### 3.4.1. How to Perform the Block

When conducting paravertebral blocks, it is essential always to be aware of potential hazards, including a small risk of pneumothorax, the unintended injection of the drug into the epidural or intrathecal space, and the faster absorption of LA [66]. The ultrasound probe is placed in a transverse position, targeting the bony structures, specifically the transverse process connecting the rib. It is crucial to differentiate the pleura, which exhibits movement with each breath. The needle is then guided between the costotransverse ligament and the pleura, and during the injection, the observation of pleural displacement is essential to confirm precise positioning.

#### 3.4.2. Evidence and Indications

Thoracic paravertebral blocks (PVBs) are as effective as thoracic epidurals for pain relief in chest wall trauma, rib fractures, post-thoracotomy cases, and situations where epidural anesthesia is not recommended [67,68]. The paravertebral block is a more straightforward procedure with fewer minor complications such as pneumothorax and, rarely, urinary retention, itching, nausea, and low blood pressure [69,70]. This makes it suitable for patients with varying degrees of hemodynamic instability. In patients with rib fractures, the paravertebral block is more effective for pain control than systemic opioid therapy [71]. After video-assisted thoracoscopic lobectomy for lung cancer, however, continuous paravertebral analgesia had significantly higher pain scores at rest and while coughing at 24 and 48 h than an epidural approach [72]. The PVB anesthetizes spinal nerves emerging from intervertebral foramina and produces unilateral, segmental, somatic, and sympathetic nerve block.

#### 3.4.3. Why the ICU Physician Could Love It

The PVB and ESPB have overlapping possible applications, and the ESPB might provide the superior safety profile in most cases. However, especially in anatomical deviations like severe scoliosis, the PVB might be easier to perform.

### 3.5. Parasternal Block

Parasternal block techniques serve as valuable methods for administering regional analgesia to the central chest region. They are suitable to complement lateral chest wall blocks or as stand-alone blocks after median sternotomy. Parasternal blocks include the pecto-intercostal fascial block (PIFB) and the Transversus Thoracic Plane Block (TTPB) [73,74]. Both blocks address the same area of action. While the PIFB is more superficial and usually due to an incomplete fascial spread, it needs two injections per site. The TTPB is a deeper technique, where one injection per site is generally sufficient at the cost of a slightly higher potential for complications due to its proximity to the pleural line. Both blocks target the sensory dermatomes T2–T6 by blocking anterior branches of the intercostal nerves to provide analgesia of the area along the sternum.

#### 3.5.1. How to Perform the Block

**Figure 4:**
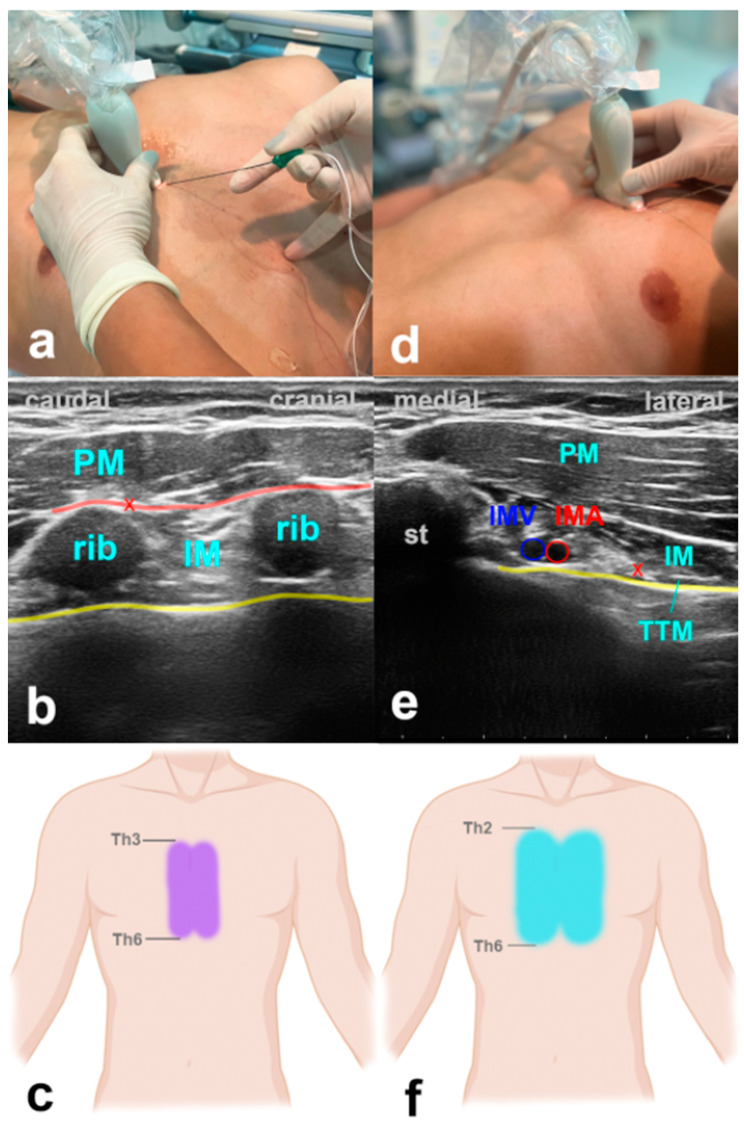
Parasternal blocks. (**a**) Transducer positioning for PIFB. (**b**) Ultrasound anatomy of PIFB: PM—pectoralis muscle; IM—intercostal muscle; yellow line—pleura; red cross—target point for the injection at the edge of the rib; red line—target space for PIFB. (**c**) Spread of PIFB. (**d**) Transducer positioning for TTPB. (**e**) Ultrasound anatomy of TTPB: PM—pectoralis muscle; IM—intercostal muscle; TTM—transverse thoracic muscle; st—sternum; IMV—internal mammary vein; IMA—internal mammary artery; yellow line—pleura; red cross—target point for the injection between IM and TTM. (**f**) Spread of TTPB.

With the patient in the supine position, for the PIFB, the ultrasound probe is positioned in a craniocaudal direction at the level of the second intercostal space about 2 cm off the sternal edge (Figure 4). USG landmarks are the pectoralis major, the internal intercostal muscle, and the ribs. The target is the fascial plane between the pectoralis major and the external intercostal muscle. The procedure is repeated at the level of the fourth intercostal space. For the TTPB, the transducer is positioned in a transverse orientation at the level of the second intercostal space. The reason for that is the need to explicitly identify the internal mammary vessels located in the neurovascular plane between the intercostal and the transverse thoracic muscle, which is the target for the LA application. Volumes of LA are indicated in Table 1.

#### 3.5.2. Evidence and Indications

Several studies provide data on parasternal blocks decreasing intra- and postoperative opioid use, time to extubation, and improved postoperative performance at spirometry in cardiac surgery patients [73,75,76,77]. A recent meta-analysis showed a significant reduction in postoperative pain and opioid use in patients after median sternotomy [78].

#### 3.5.3. Why the ICU Physician Could Love It

There is currently no alternative to these blocks other than intravenous analgesics. USG parasternal blocks offer an easy-to-perform, fast, and reliable possibility to control unexpected or insufficiently controlled post-sternotomy pain. Mainly, when performed after a CABG procedure with the harvesting of the mammary artery due to the altered anatomy, the PIFB might provide advantages in feasibility over the TTPB.

### 3.6. Intercostal Block

Intercostal blocks have been shown to offer reasonable pain control for invasive procedures such as chest tube insertion or in patients with rib fractures [79,80,81]. Compared to neuraxial methods or the ESPB, a significant disadvantage is the need for several punctures when targeting multiple levels, and some studies have yielded inferior performance regarding pain control compared to paravertebral or epidural block [70]. When performing intercostal block for pain control in rib fractures, it is recommended to perform the block at each level of costal fracture plus one level below and above to ensure complete coverage. Consequently, a minimum of three punctures would be needed in the case of one fractured rib, which might make the ESPB the favorable option. 

## 4. Peripheral Nerve Blocks for the Abdominal Wall

### 4.1. Transversus Abdominis Plane Block

#### 4.1.1. How to Perform the Block

**Figure 5:**
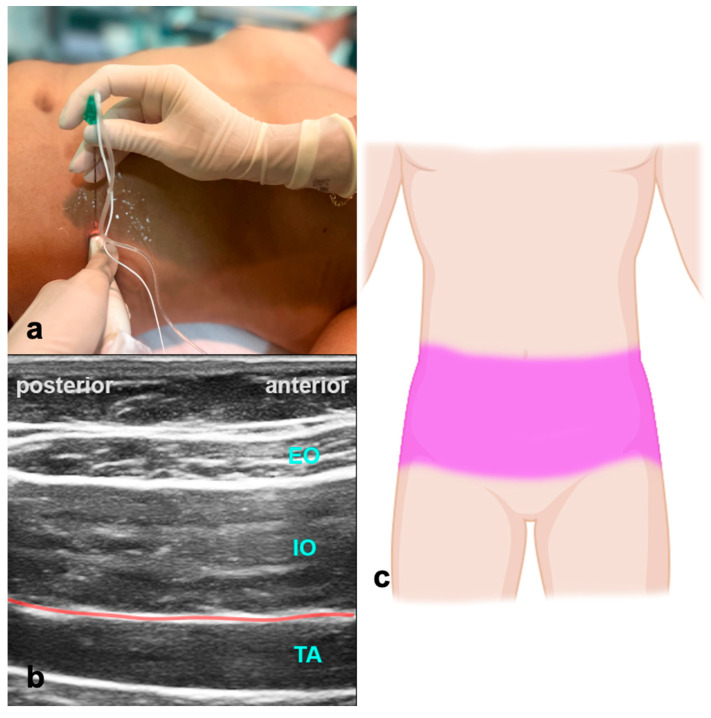
Transversus abdominis plane block. (**a**) Transducer positioning for TAPB. (**b**) Ultrasound anatomy of TAPB: EO—external oblique muscle; IO—internal oblique muscle; TA—transverse abdominal muscle; red line—target space for TAPB. (**c**) Spread of TAPB if applied bilaterally.

The patient is in a supine position. The ultrasound probe is placed transverse to the abdominal wall between the costal arch and the iliac crest in the mid-axillary line (Figure 5). USG identification marks are the external oblique, internal oblique, and transversus abdominis muscle. The LA is applied between the internal oblique and transversus abdominis muscle, providing analgesia of the abdominal wall up to Th10. Volumes of LA are indicated in Table 1. The block can be performed bilaterally for procedures with midline incisions. When doing this, care should be taken not to exceed the maximum dose of LA.

#### 4.1.2. Evidence and Indications

The transversus abdominis plane block (TAPB) provides effective postoperative analgesia for various procedures involving the abdominal wall [82,83,84]. In the EXPLANE trial, postoperative pain scores were comparable in patients treated with the TAPB compared to epidural analgesia in abdominal surgery patients. Patients with the TAPB needed slightly more opioids but had significantly less hypotension [85]. Compared to intravenous opioid-based strategies, the TAPB improved perioperative pain management in multiple abdominal procedures [86,87,88]. A recent meta-analysis found the TAPB and ESPB to be equally effective [89]. One case series presented the feasibility and safety of a postoperative TAPB in ICU patients. 

#### 4.1.3. Why the ICU Physician Could Love It

Major abdominal procedures are common and often necessitate substantial intravenous pain medications, each carrying side effects that can impact patient outcomes. Transversus abdominis plane blocks offer a straightforward, safe alternative without requiring significant compromise to anticoagulation. Additionally, TAPBs eliminate the risk of hypotension, a common issue in the ICU and a primary reason for discontinuing epidural infusions.

### 4.2. Quadratus Lumborum Block

To the best of our knowledge, no studies are available investigating the Quadratus Lumborum Block (QLB) in an ICU population. In the perioperative setting, the QLB has been extensively studied in obstetrics, urological, gastrical, and spine surgery [90,91]. The QLB is considered an extension of the TAPB. Due to its coverage of both surface somatic and visceral pain, the QLB offers a broader spectrum of pain relief, extending the duration of relief and reducing the need for pain medication [92]. There are three Quadratus Lumborum Block (QLB) approaches described in the literature. While the lateral approach is quite similar to a TAPB, the anterior approach targets the intermuscular fascial plane between the psoas and the quadratus lumborum muscle to block the intercostal nerves of the abdominal wall at this level, thereby facilitating a more comprehensive block of the abdominal wall compared to the TAPB. The QLB is a highly efficient regional anesthesia technique renowned for its extensive blocking range, prolonged pain relief, and outstanding pain management outcomes. However, it may be technically more demanding, and its effectiveness seems to depend on the chosen approach [93].

### 4.3. Rectus Sheath Block and Subcostal Tap Block

The rectus sheath block and the subcostal tap block are valuable options as well. They may be more applicable in open abdominal surgery when compared to the TAPB. However, the typical approach will usually be postoperatively covered by sterile wound dressings, thereby limiting their applicability. 

## 5. Conclusions

Mono-modal pain management strategies centered around opioids have demonstrated shortcomings in adequately controlling pain for a majority of ICU patients. In contrast, multi-modal approaches have proven to be significantly more effective in enhancing pain control in these patients. Recent advancements in the easy-to-perform PNB and FPB have expanded the scope of regional anesthesia, offering considerable value in the context of these preferred multi-modal strategies. Importantly, many of these blocks exhibit favorable safety profiles when compared to neuraxial procedures, while still making substantial contributions to pain management. This improved risk–benefit ratio makes PNBs/FPBs valuable tools for ICU physicians, especially in cases where neuraxial blocks are either undesired or contraindicated. However, the majority of evidence comes from the perioperative setting, and future studies should address the role of peripheral RA techniques in ICU patients.

## Figures and Tables

**Table 1:** Recommendations of local anesthetics and dosages for PNB/FPB.

**PNB/FPB**	**Dosage Recommendation**
PECS I	Bupivacaine 0.25% or Ropivacaine 0.5%: 10 mL
PECS II	Bupivacaine 0.25% or Ropivacaine 0.5%: 20 mL
SAPB	Bupivacaine 0.25%: 30–40 mL Continuous Catheter Infusion: Ropivacaine 0.2%: 6–10 mL/h; Bolus 8 mL; Lockout interval 30 min
ESPB	Bupivacaine 0.25% or Ropivacaine 0.5% Unilateral: 50–70 kg: 30 mL; ≥70 kg: 40 mL Bilateral (each side): 50–70 kg: 20 mL; ≥70–100 kg: 25 mL; ≥100 kg: 30 mL Continuous Catheter Infusion: Ropivacaine 0.2%: 8–10 mL/h; Bolus 5 mL, Lockout interval 60 min Alternative: Intermittent Bolus of 15 mL each 2–3 h
PIFB/TTPB	Bupivacaine 0.25% or Ropivacaine 0.5%: 20 mL/side
Intercostal Block	Ropivacaine 0.2%: 3–5 mL per level
TAPB	Bupivacaine 0.25% or Ropivacaine 0.5%: 20 mL Continuous Catheter Infusion: Ropivacaine 0.2%: 6–10 mL/h; Bolus 12 mL; Lockout interval 60 min

PNB—paravertebral nerve block; FPB—fascial plane block; PECS—pectoralis nerve block; SAPB—serratus anterior plane block; EPSB—erector spinae plane block; PIFB—pecto-intercostal fascial block; TTPB—Transversus Thoracic Plane Block; TAPB—transversus abdominis plane block. Dosage recommendations according local standards.

## Data Availability

No new data were created or analyzed in this study. Data sharing is not applicable to this article.

## References

[1] Stanik-Hutt J.A., Soeken K.L., Belcher A.E., Fontaine D.K., Gift A.G. (2001). Pain experiences of traumatically injured patients in a critical care setting. Am. J. Crit. Care.

[2] Chanques G., Sebbane M., Barbotte E., Viel E., Eledjam J.J., Jaber S. (2007). A prospective study of pain at rest: Incidence and characteristics of an unrecognized symptom in surgical and trauma versus medical intensive care unit patients. Anesthesiology.

[3] Moliner Velázquez S., Rubio Haro R., De Andrés Serrano C., De Andrés Ibáñez J. (2017). Regional analgesia in postsurgical critically ill patients. Rev. Esp. Anestesiol. Reanim..

[4] Ehieli E., Yalamuri S., Brudney C.S., Pyati S. (2017). Analgesia in the surgical intensive care unit. Postgrad. Med. J..

[5] Turan A., Leung S., Bajracharya G.R., Babazade R., Barnes T., Schacham Y.N., Mao G., Zimmerman N., Ruetzler K., Maheshwari K. (2020). Acute Postoperative Pain Is Associated With Myocardial Injury After Noncardiac Surgery. Anesth. Analg..

[6] Baratta J.L., Schwenk E.S., Viscusi E.R. (2014). Clinical Consequences of Inadequate Pain Relief: Barriers to Optimal Pain Management. Plast. Reconstr. Surg..

[7] McGuire L., Heffner K., Glaser R., Needleman B., Malarkey W., Dickinson S., Lemeshow S., Cook C., Muscarella P., Melvin W.S. (2006). Pain and wound healing in surgical patients. Ann. Behav. Med..

[8] Joshi G.P., Ogunnaike B.O. (2005). Consequences of inadequate postoperative pain relief and chronic persistent postoperative pain. Anesthesiol. Clin. North. Am..

[9] Baumbach P., Götz T., Günther A., Weiss T., Meissner W. (2016). Prevalence and Characteristics of Chronic Intensive Care-Related Pain: The Role of Severe Sepsis and Septic Shock. Crit. Care Med..

[10] Battle C.E., Lovett S., Hutchings H. (2013). Chronic pain in survivors of critical illness: A retrospective analysis of incidence and risk factors. Crit. Care.

[11] Korosec Jagodic H., Jagodic K., Podbregar M. (2006). Long-term outcome and quality of life of patients treated in surgical intensive care: A comparison between sepsis and trauma. Crit. Care.

[12] Kalso E., Mennander S., Tasmuth T., Nilsson E. (2001). Chronic post-sternotomy pain. Acta Anaesthesiol. Scand..

[13] Eisenberg E., Pultorak Y., Pud D., Bar-El Y. (2001). Prevalence and characteristics of post coronary artery bypass graft surgery pain (PCP). Pain.

[14] Drahos A.L., Scott A.M., Johns T.J., Ashley D.W. (2020). Multimodal Analgesia and Decreased Opioid Use in Adult Trauma Patients. Am. Surg..

[15] Gausche-Hill M., Brown K.M., Oliver Z.J., Sasson C., Dayan P.S., Eschmann N.M., Weik T.S., Lawner B.J., Sahni R., Falck-Ytter Y. (2014). An Evidence-based Guideline for Prehospital Analgesia in Trauma. Prehospital Emerg. Care.

[16] Jacobi J., Fraser G.L., Coursin D.B., Riker R.R., Fontaine D., Wittbrodt E.T., Chalfin D.B., Masica M.F., Bjerke H.S., Coplin W.M. (2002). Clinical practice guidelines for the sustained use of sedatives and analgesics in the critically ill adult. Crit. Care Med..

[17] Devlin J.W., Skrobik Y., Gélinas C., Needham D.M., Slooter A.J.C., Pandharipande P.P., Watson P.L., Weinhouse G.L., Nunnally M.E., Rochwerg B. (2018). Clinical Practice Guidelines for the Prevention and Management of Pain, Agitation/Sedation, Delirium, Immobility, and Sleep Disruption in Adult Patients in the ICU. Crit. Care Med..

[18] Chou R., Gordon D.B., de Leon-Casasola O.A., Rosenberg J.M., Bickler S., Brennan T., Carter T., Cassidy C.L., Chittenden E.H., Degenhardt E. (2016). Management of Postoperative Pain: A Clinical Practice Guideline From the American Pain Society, the American Society of Regional Anesthesia and Pain Medicine, and the American Society of Anesthesiologists’ Committee on Regional Anesthesia, Executive Committee, and Administrative Council. J. Pain.

[19] Kohler M., Chiu F., Gelber K.M., Webb C.A., Weyker P.D. (2016). Pain management in critically ill patients: A review of multimodal treatment options. Pain Manag..

[20] Xiao D.L., Xi J.W. (2023). Efficacy of peripheral nerve blocks for pain management in patients with rib fractures: A systematic review and meta-analysis. Eur. Rev. Med. Pharmacol. Sci..

[21] De Pinto M., Dagal A., O’Donnell B., Stogicza A., Chiu S., Edwards W.T. (2015). Regional anesthesia for management of acute pain in the intensive care unit. Int. J. Crit. Illn. Inj. Sci..

[22] Saasouh W., Turan A. (2017). Ultrasound a Game Changer. Turk. J. Anaesthesiol. Reanim..

[23] Venkataraju A., Bhatia K. (2013). Practice of regional anaesthesia in critical care units in the North West England. Reg. Anesth. Pain. Med..

[24] Venkataraju A., Narayanan M. (2016). Analgesia in intensive care: Part 2. BJA Educ..

[25] Casati A., Baciarello M., Di Cianni S., Danelli G., De Marco G., Leone S., Rossi M., Fanelli G. (2007). Effects of ultrasound guidance on the minimum effective anaesthetic volume required to block the femoral nerve. Br. J. Anaesth..

[26] Visser W.A., Gielen M.J., Giele J.L. (2006). Continuous positive airway pressure breathing increases the spread of sensory blockade after low-thoracic epidural injection of lidocaine. Anesth. Analg..

[27] Joshi G., Gandhi K., Shah N., Gadsden J., Corman S.L. (2016). Peripheral nerve blocks in the management of postoperative pain: Challenges and opportunities. J. Clin. Anesth..

[28] Hebl J.R., Niesen A.D. (2011). Infectious complications of regional anesthesia. Curr. Opin. Anaesthesiol..

[29] Cook T.M., Counsell D., Wildsmith J.A. (2009). Major complications of central neuraxial block: Report on the Third National Audit Project of the Royal College of Anaesthetists. Br. J. Anaesth..

[30] Alshahrani K.M., Alhuwaishel A.Z., Alangari N.M., Asiri M.A., Al-Shahrani N.A., Alasmari A.A., Alzahrani O.J., Ayedh A.Y., Qitmah M.M. (2023). Clinical Impacts and Risk Factors for Central Line-Associated Bloodstream Infection: A Systematic Review. Cureus.

[31] Sun Q., Li Z., Wang Z., Wang Q., Qin F., Pan H., Lin W., Mu X., Wang Y., Jiang Y. (2023). Immunosuppression by opioids: Mechanisms of action on innate and adaptive immunity. Biochem. Pharmacol..

[32] Martyn J.A.J., Mao J., Bittner E.A. (2019). Opioid Tolerance in Critical Illness. N. Engl. J. Med..

[33] Horlocker T.T., Vandermeuelen E., Kopp S.L., Gogarten W., Leffert L.R., Benzon H.T. (2018). Regional Anesthesia in the Patient Receiving Antithrombotic or Thrombolytic Therapy: American Society of Regional Anesthesia and Pain Medicine Evidence-Based Guidelines (Fourth Edition). Reg. Anesth. Pain Med..

[34] El-Sherbiny S.M., Kamal R.A., Elhadary I.H., Abdallah M.Y.Y. (2022). Erector spinae plane block versus thoracic epidural block as analgesic techniques for chest trauma: A randomized controlled trial. Res. Opin. Anesth. Intensive Care.

[35] Pintaric T.S., Potocnik I., Hadzic A., Stupnik T., Pintaric M., Novak Jankovic V. (2011). Comparison of continuous thoracic epidural with paravertebral block on perioperative analgesia and hemodynamic stability in patients having open lung surgery. Reg. Anesth. Pain Med..

[36] Kaur U., Shamshery C., Agarwal A., Prakash N., Valiveru R.C., Mishra P. (2020). Evaluation of postoperative pain in patients undergoing modified radical mastectomy with pectoralis or serratus-intercostal fascial plane blocks. Korean J. Anesthesiol..

[37] Abu Elyazed M.M., Abdelghany M.S., Mostafa S.F. (2020). The Analgesic Efficacy of Pecto-Intercostal Fascial Block Combined with Pectoral Nerve Block in Modified Radical Mastectomy: A Prospective Randomized Trial. Pain Physician.

[38] Barrington M.J., Seah G.J., Gotmaker R., Lim D., Byrne K. (2020). Quality of Recovery After Breast Surgery: A Multicenter Randomized Clinical Trial Comparing Pectoral Nerves Interfascial Plane (Pectoral Nerves II) Block With Surgical Infiltration. Anesth. Analg..

[39] Alfirevic A., Marciniak D., Duncan A.E., Kelava M., Yalcin E.K., Hamadnalla H., Pu X., Sessler D.I., Bauer A., Hargrave J. (2023). Serratus anterior and pectoralis plane blocks for robotically assisted mitral valve repair: A randomised clinical trial. Br. J. Anaesth..

[40] Hoerner E., Stundner O., Naegele F., Fiala A., Bonaros N., Mair P., Holfeld J., Gasteiger L. (2023). The impact of PECS II blockade in patients undergoing minimally invasive cardiac surgery-a prospective, randomized, controlled, and triple-blinded trial. Trials.

[41] Durant E., Dixon B., Luftig J., Mantuani D., Herring A. (2017). Ultrasound-guided serratus plane block for ED rib fracture pain control. Am. J. Emerg. Med..

[42] Park M.H., Kim J.A., Ahn H.J., Yang M.K., Son H.J., Seong B.G. (2018). A randomised trial of serratus anterior plane block for analgesia after thoracoscopic surgery. Anaesthesia.

[43] Blanco R., Parras T., McDonnell J.G., Prats-Galino A. (2013). Serratus plane block: A novel ultrasound-guided thoracic wall nerve block. Anaesthesia.

[44] Piracha M.M., Thorp S.L., Puttanniah V., Gulati A. (2017). “A Tale of Two Planes”: Deep Versus Superficial Serratus Plane Block for Postmastectomy Pain Syndrome. Reg. Anesth. Pain Med..

[45] Bhalla P.I., Solomon S., Zhang R., Witt C.E., Dagal A., Joffe A.M. (2021). Comparison of serratus anterior plane block with epidural and paravertebral block in critically ill trauma patients with multiple rib fractures. Trauma. Surg. Acute Care Open.

[46] Diwan S., Nair A. (2021). A retrospective study comparing analgesic efficacy of ultrasound-guided serratus anterior plane block versus intravenous fentanyl infusion in patients with multiple rib fractures. J. Anaesthesiol. Clin. Pharmacol..

[47] Liu X., Song T., Xu H.Y., Chen X., Yin P., Zhang J. (2020). The serratus anterior plane block for analgesia after thoracic surgery: A meta-analysis of randomized controlled trails. Medicine.

[48] Khalil A.E., Abdallah N.M., Bashandy G.M., Kaddah T.A. (2017). Ultrasound-Guided Serratus Anterior Plane Block Versus Thoracic Epidural Analgesia for Thoracotomy Pain. J. Cardiothorac. Vasc. Anesth..

[49] Elsabeeny W.Y., Ibrahim M.A., Shehab N.N., Mohamed A., Wadod M.A. (2021). Serratus Anterior Plane Block and Erector Spinae Plane Block Versus Thoracic Epidural Analgesia for Perioperative Thoracotomy Pain Control: A Randomized Controlled Study. J. Cardiothorac. Vasc. Anesth..

[50] Statzer N.J., Plackis A.C., Woolard A.A., Allen B.F.S., Siegrist K.K., Shi Y., Shotwell M. (2022). Erector Spinae Plane Catheter Analgesia in Minimally Invasive Mitral Valve Surgery: A Retrospective Case-Control Study for Inclusion in an Enhanced Recovery Program. Semin. Cardiothorac. Vasc. Anesth..

[51] Nagaraja P.S., Ragavendran S., Singh N.G., Asai O., Bhavya G., Manjunath N., Rajesh K. (2018). Comparison of continuous thoracic epidural analgesia with bilateral erector spinae plane block for perioperative pain management in cardiac surgery. Ann. Card. Anaesth..

[52] Ragavendran S., Raghu C., Prasad S.R., Arasu T., Nagaraja P.S., Singh N.G., Manjunath N., Muralikrishna N., Yogananth N. (2022). Comparison of epidural analgesia with ultrasound-guided bilateral erector spinae plane block in aorto-femoral arterial bypass surgery. Ann. Card. Anaesth..

[53] Sørenstua M., Zantalis N., Raeder J., Vamnes J.S., Leonardsen A.L. (2023). Spread of local anesthetics after erector spinae plane block: An MRI study in healthy volunteers. Reg. Anesth. Pain Med..

[54] Toscano A., Capuano P., Costamagna A., Canavosio F.G., Ferrero D., Alessandrini E.M., Giunta M., Rinaldi M., Brazzi L. (2022). Is continuous Erector Spinae Plane Block (ESPB) better than continuous Serratus Anterior Plane Block (SAPB) for mitral valve surgery via mini-thoracotomy? Results from a prospective observational study. Ann. Card. Anaesth..

[55] Finnerty D.T., McMahon A., McNamara J.R., Hartigan S.D., Griffin M., Buggy D.J. (2020). Comparing erector spinae plane block with serratus anterior plane block for minimally invasive thoracic surgery: A randomised clinical trial. Br. J. Anaesth..

[56] Nair A., Saxena P., Borkar N., Rangaiah M., Arora N., Mohanty P.K. (2023). Erector spinae plane block for postoperative analgesia in cardiac surgeries—A systematic review and meta-analysis. Ann. Card. Anaesth..

[57] Morkos M., DeLeon A., Koeckert M., Gray Z., Liao K., Pan W., Tolpin D.A. (2023). The Use of Unilateral Erector Spinae Plane Block in Minimally Invasive Cardiac Surgery. J. Cardiothorac. Vasc. Anesth..

[58] Krishna S.N., Chauhan S., Bhoi D., Kaushal B., Hasija S., Sangdup T., Bisoi A.K. (2019). Bilateral Erector Spinae Plane Block for Acute Post-Surgical Pain in Adult Cardiac Surgical Patients: A Randomized Controlled Trial. J. Cardiothorac. Vasc. Anesth..

[59] D’Hondt N., Rex S., Verbrugghe P., Van den Eynde R., Hoogma D. (2020). Erector spinae plane block for enhanced recovery after cardiac surgery in minimally invasive mitral valve surgery. J. Cardiothorac. Vasc. Anesth..

[60] Wiech M., Żurek S., Kurowicki A., Horeczy B., Czuczwar M., Piwowarczyk P., Widenka K., Borys M. (2022). Erector Spinae Plane Block Decreases Chronic Postoperative Pain Severity in Patients Undergoing Coronary Artery Bypass Grafting. J. Clin. Med..

[61] Syal R., Mohammed S., Kumar R., Jain N., Bhatia P. (2021). Continuous erector spinae plane block for analgesia and better pulmonary functions in patients with multiple rib fractures: A prospective descriptive study. Braz. J. Anesthesiol. (Engl. Ed.).

[62] Hamilton D.L., Manickam B. (2017). Erector spinae plane block for pain relief in rib fractures. Br. J. Anaesth..

[63] Elshazly M., El-Halafawy Y.M., Mohamed D.Z., Wahab K.A.E., Mohamed T.M.K. (2022). Feasibility and efficacy of erector spinae plane block versus transversus abdominis plane block in laparoscopic bariatric surgery: A randomized comparative trial. Korean J. Anesthesiol..

[64] Wan F.T., Chin S.E., Gwee R., Chong Y., Au-Yong A., Matthews A., Zaw M.W., Lie S.A., Loh L., Koh D. (2023). Pre-operative erector spinae plane block should be considered a viable option for laparoscopic colectomies. Surg. Endosc..

[65] Kelava M., Anthony D., Elsharkawy H. (2018). Continuous Erector Spinae Block for Postoperative Analgesia After Thoracotomy in a Lung Transplant Recipient. J. Cardiothorac. Vasc. Anesth..

[66] Schnabel A., Reichl S.U., Kranke P., Pogatzki-Zahn E.M., Zahn P.K. (2010). Efficacy and safety of paravertebral blocks in breast surgery: A meta-analysis of randomized controlled trials. Br. J. Anaesth..

[67] Mohta M., Verma P., Saxena A.K., Sethi A.K., Tyagi A., Girotra G. (2009). Prospective, randomized comparison of continuous thoracic epidural and thoracic paravertebral infusion in patients with unilateral multiple fractured ribs--a pilot study. J. Trauma..

[68] Casati A., Alessandrini P., Nuzzi M., Tosi M., Iotti E., Ampollini L., Bobbio A., Rossini E., Fanelli G. (2006). A prospective, randomized, blinded comparison between continuous thoracic paravertebral and epidural infusion of 0.2% ropivacaine after lung resection surgery. Eur. J. Anaesthesiol..

[69] Karmakar M.K., Greengrass R.A., Latmore M., Levin M., Hadzic A. (2017). Thoracic & Lumbar Paravertebral Block. Hadzic’s Textbook of Regional Anesthesia and Acute Pain Management.

[70] Joshi G.P., Bonnet F., Shah R., Wilkinson R.C., Camu F., Fischer B., Neugebauer E.A., Rawal N., Schug S.A., Simanski C. (2008). A systematic review of randomized trials evaluating regional techniques for postthoracotomy analgesia. Anesth. Analg..

[71] Malekpour M., Hashmi A., Dove J., Torres D., Wild J. (2017). Analgesic Choice in Management of Rib Fractures: Paravertebral Block or Epidural Analgesia?. Anesth. Analg..

[72] Lai J., Situ D., Xie M., Yu P., Wang J., Long H., Lai R. (2021). Continuous Paravertebral Analgesia versus Continuous Epidural Analgesia after Video-Assisted Thoracoscopic Lobectomy for Lung Cancer: A Randomized Controlled Trial. Ann. Thorac. Cardiovasc. Surg..

[73] Zhang Y., Gong H., Zhan B., Chen S. (2021). Effects of bilateral Pecto-intercostal Fascial Block for perioperative pain management in patients undergoing open cardiac surgery: A prospective randomized study. BMC Anesthesiol..

[74] Desire S.M., Hayward G. (2023). Transversus Thoracic Muscle Plane Block (TTMPB). StatPearls.

[75] Schiavoni L., Nenna A., Cardetta F., Pascarella G., Costa F., Chello M., Agrò F.E., Mattei A. (2022). Parasternal Intercostal Nerve Blocks in Patients Undergoing Cardiac Surgery: Evidence Update and Technical Considerations. J. Cardiothorac. Vasc. Anesth..

[76] Pascarella G., Costa F., Nonnis G., Strumia A., Sarubbi D., Schiavoni L., Di Pumpo A., Mortini L., Grande S., Attanasio A. (2023). Ultrasound Guided Parasternal Block for Perioperative Analgesia in Cardiac Surgery: A Prospective Study. J. Clin. Med..

[77] Chen H., Song W., Wang W., Peng Y., Zhai C., Yao L., Xia Z. (2021). Ultrasound-guided parasternal intercostal nerve block for postoperative analgesia in mediastinal mass resection by median sternotomy: A randomized, double-blind, placebo-controlled trial. BMC Anesthesiol..

[78] King M., Stambulic T., Hassan S.M.A., Norman P.A., Derry K., Payne D.M., El Diasty M. (2022). Median sternotomy pain after cardiac surgery: To block, or not? A systematic review and meta-analysis. J. Card. Surg..

[79] Luketich J.D., Land S.R., Sullivan E.A., Alvelo-Rivera M., Ward J., Buenaventura P.O., Landreneau R.J., Hart L.A., Fernando H.C. (2005). Thoracic epidural versus intercostal nerve catheter plus patient-controlled analgesia: A randomized study. Ann. Thorac. Surg..

[80] De Pinto M., Edwards W. (2016). Management of Pain in the Critically Ill Patient in Irwin-Rippe’s Intensive Care Medicine.

[81] Britt T., Sturm R., Ricardi R., Labond V. (2015). Comparative evaluation of continuous intercostal nerve block or epidural analgesia on the rate of respiratory complications, intensive care unit, and hospital stay following traumatic rib fractures: A retrospective review. Local Reg. Anesth..

[82] Carney J., McDonnell J.G., Ochana A., Bhinder R., Laffey J.G. (2008). The transversus abdominis plane block provides effective postoperative analgesia in patients undergoing total abdominal hysterectomy. Anesth. Analg..

[83] McDonnell J.G., Curley G., Carney J., Benton A., Costello J., Maharaj C.H., Laffey J.G. (2008). The analgesic efficacy of transversus abdominis plane block after cesarean delivery: A randomized controlled trial. Anesth. Analg..

[84] Belavy D., Cowlishaw P.J., Howes M., Phillips F. (2009). Ultrasound-guided transversus abdominis plane block for analgesia after Caesarean delivery. Br. J. Anaesth..

[85] Turan A., Cohen B., Elsharkawy H., Maheshwari K., Soliman L.M., Babazade R., Ayad S., Hassan M., Elkassabany N., Essber H.A. (2022). Transversus abdominis plane block with liposomal bupivacaine versus continuous epidural analgesia for major abdominal surgery: The EXPLANE randomized trial. J. Clin. Anesth..

[86] Niraj G., Kelkar A., Hart E., Kaushik V., Fleet D., Jameson J. (2015). Four quadrant transversus abdominis plane block and continuous transversus abdominis plane analgesia: A 3-year prospective audit in 124 patients. J. Clin. Anesth..

[87] Niraj G., Kelkar A., Jeyapalan I., Graff-Baker P., Williams O., Darbar A., Maheshwaran A., Powell R. (2011). Comparison of analgesic efficacy of subcostal transversus abdominis plane blocks with epidural analgesia following upper abdominal surgery. Anaesthesia.

[88] Fusco P., Scimia P., Paladini G., Fiorenzi M., Petrucci E., Pozone T., Vacca F., Behr A., Micaglio M., Danelli G. (2015). Transversus abdominis plane block for analgesia after Cesarean delivery. A systematic review. Minerva Anestesiol..

[89] Liheng L., Siyuan C., Zhen C., Changxue W. (2022). Erector Spinae Plane Block versus Transversus Abdominis Plane Block for Postoperative Analgesia in Abdominal Surgery: A Systematic Review and Meta-Analysis. J. Investig. Surg..

[90] Uppal V., Retter S., Kehoe E., McKeen D.M. (2020). Quadratus lumborum block for postoperative analgesia: A systematic review and meta-analysis. Can. J. Anaesth..

[91] Kim S.H., Kim H.J., Kim N., Lee B., Song J., Choi Y.S. (2020). Effectiveness of quadratus lumborum block for postoperative pain: A systematic review and meta-analysis. Minerva Anestesiol..

[92] Wang Y., Wang X., Zhang K. (2020). Effects of transversus abdominis plane block versus quadratus lumborum block on postoperative analgesia: A meta-analysis of randomized controlled trials. BMC Anesthesiol..

[93] Long X., Yin Y., Guo W., Tang L. (2023). Ultrasound-guided quadratus lumborum block: A powerful way for reducing postoperative pain. Ann. Med. Surg..

